# Overcoming personal information protection challenges involving real-world data to support public health efforts in China

**DOI:** 10.3389/fpubh.2023.1265050

**Published:** 2023-09-22

**Authors:** Yu Yao, Fei Yang

**Affiliations:** School of Law, China Jiliang University, Hangzhou, China

**Keywords:** personal information, health data, cross-border data transfer, privacy, real world data

## Abstract

In the information age, real-world data-based evidence can help extrapolate and supplement data from randomized controlled trials, which can benefit clinical trials and drug development and improve public health decision-making. However, the legitimate use of real-world data in China is limited due to concerns over patient confidentiality. The use of personal information is a core element of data governance in public health. In China’s public health data governance, practical problems exist, such as balancing personal information protection and public value conflict. In 2021, China adopted the Personal Information Protection Law (PIPL) to provide a consistent legal framework for protecting personal information, including sensitive medical health data. Despite the PIPL offering critical legal safeguards for processing health data, further clarification is needed regarding specific issues, including the meaning of “separate consent,” cross-border data transfer requirements, and exceptions for scientific research. A shift in the law and regulatory framework is necessary to advance public health research further and realize the potential benefits of combining real-world evidence and digital health while respecting privacy in the technological and demographic change era.

## 1. Introduction

Despite the absence of a globally agreed-upon definition in recent years, the term *real-world data* (RWD) quickly morphed into a buzzword (1). Definitions of RWD differ depending on the source or environment from which the data were generated or collected (2). Given its enhanced popularity, China’s National Medical Products Administration (NMPA) introduced a series of policies to standardize and guide the application of RWD. Per the NMPA’s definition, RWD are “various data related to patients’ health status and/or diagnosis and health care that are collected on a daily basis, opposed to data gathered in experimental settings such as randomized controlled trials” (3). RWD contains digital and non-digital data from various sources, including hospital electronic medical records (EMRs), classical cohort studies, administrative databases, daily wearable devices, and surveillance databases (4). Using RWD to inform health-related decisions is referred to as real-world evidence (RWE). NMPA further defines RWE as “the clinical evidence about the use and potential benefit–risk of a drug obtained through appropriate and adequate analysis of applicable RWD.” This definition can be used to support drug development and regulatory decisions and for other scientific purposes (3, 5).

The RWD-based study (RWS) (6, 7) has been frequently used to address recent public health issues. This is because addressing urgent public health requirements today requires access to and cross-referencing of several types of data (8). For example, the COVID-19 pandemic demonstrated that promptly capturing and using RWD from community testing can support real-time monitoring and decision-making by public health agencies (9). When threats to public health arise from epidemic outbreaks, government agencies should have access to RWD from various sources like EMRs, pharmacy claims, medical claims, and labs to understand disease spread, predict potential outbreaks, create surveillance systems, allocate resources, and make informed decisions (10). RWS is characterized by a vast research population, diverse study scenarios, substantial heterogeneity among a wide range of data sources, and high external validity. Other RWS applications to address public health issues can be found in the literature, such as a scoping review that demonstrated potential for applying RWD to address noncommunicable diseases with greater precision (11). Recently, Sarovar et al. (12) conducted a study using air pollution and fetal death certificate data to provide evidence for associations between prenatal short-term air pollution exposure and stillbirth.

In China, Du (13) first introduced the concept of RWS, describing its use in evaluating the safety of Traditional Chinese Medicine injections. In response to the increasing interest in conducting RWS, the NMPA organized representatives from academia, the pharmaceutical industry, and relevant institutions to formulate policies for using RWD in the medical and healthcare field and to promote RWS-based regulatory science (14). Chinese researchers have made efforts in the field of RWS related to public health research. Yin et al. (15) conducted a RWS using EMRs collected on 1,446 inpatients with neurosurgery operations in a grade A tertiary hospital during a 5-year period to address the public health threat from antibiotic resistance. On June 9, 2023, China launched a RWS on COVID-19-listed drugs in Beijing. Data from nearly 40,000 patients at more than 100 large and grassroots hospitals across 30 provinces, cities, and autonomous regions will be included in the RWS (16). This RWS will provide more solid evidence for COVID-19 drugs’ efficacy, effectiveness, and safety, and assist in validating their use in clinical settings. Moreover, this RWS is an essential exercise for China to strengthen the construction of the public health emergency system at the grassroots level.

Real-world data-based study have tremendous potential to promote medical research, regulatory decision-making, and public health in China. However, a few challenges remain (17–20), including inconsistent terminology and non-standardized coding, a lack of longitudinal data, an inability to process and validate data transparently, and barriers to medical health data (MHDs) access, sharing, and linkage across research institutions (21–25). This article focuses only on the challenges raised by the data protection law. The processing of RWD triggers patient privacy concerns and, therefore, must adhere to relevant data laws and regulations (26). Consequently, researchers conducting RWS in China face significant compliance challenges related to personal information (PI) protection, which generate possible legal impediments. This article suggests how best to handle these obstacles and overcome them.

## 2. The possibility of legitimate RWD processing

China’s Personal Information Protection Law (PIPL), enacted in November 2021, defines PI as “various information related to identified or identifiable natural persons recorded electronically or by other means.” It distinguishes between PI and sensitive PI and constructs a two-layered (a risk-based general clause + an opened list) style (27) criteria to identify sensitive PI (Table 1). Unlike the European Union’s (EU) General Data Protection Regulation (GDPR), which adopted both an objective context-based and a subjective purposeful approach to determine if information included sensitive data (28), the PIPL takes a consequentialist approach. The PI disclosure risk and its potential damages play decisive roles in recognizing sensitive characteristics.

**Table 1:** Criteria for identifying sensitive PI (article 28 PIPL).

Risk-based general clause	Opened list
Once disclosed or used illegally, the data may easily infringe on an individual’s dignity or harm their personal or property security.	Includes but is not limited to biometric characteristics, religious beliefs, specific identity, financial accounts, medical health, and any PI relating to minors under 14.

PI, Personal information.

Real-world data related to MHDs falls within the scope of sensitive PI. Individuals could be at risk of data exposure without appropriate legislative measures to protect these data (29). In general, the GDPR forbids processing “special categories of personal data,” including MHDs, but preserves certain exemptions to the prohibition. Unlike the GDPR, the PIPL does not prohibit the processing of sensitive PI; instead, it provides stricter duties for PI processors. Accordingly, acceptable RWD processing requires either individualized “separate” consent from each person connected with the data or anonymized PI or the research must possess a specific purpose, be sufficiently necessary, implement strict protective measures, and conduct a protection impact assessment (PIA).

## 3. Legal obstacles presented by personal information protection law for RWS in China

### 3.1. Status quo faced by RWS researchers

An inherent challenge of any data-related research involves balancing data privacy protection with data availability, especially MHDs that are highly sensitive in nature (30). Overly strict or unclear data protection rules hinder data access and sharing. Therefore, they reduce the quality and availability of data in RWS. For Chinese researchers, the particularity of China’s medical environment increases the difficulty of accessing, sharing, and linking MHDs. For example, as mentioned above, the lack of longitudinal follow-up data is an obstacle to conduct RWS. This is owing to the fact that patients often seek treatment in multiple hospitals (not through a referral system), which makes it difficult for medical institutions to obtain complete EMRs for the patient (24). To make use of longitudinal RWD, integrating fragmented data from various sources and breaking down data silos while ensuring patients’ PI security is urgently required (31). Although research institutions and technology companies have attempted to build databases and integrate patients’ EMRs and electronic health records across multiple institutions into one system (32), they face serious compliance risks under the PIPL. Since China’s PIPL does not provide for scientific research exemptions for the reuse of MHDs, the utilization and reuse of RWD should strictly comply with the PIPL, including obtaining re-consent from the patients. Data fragmentation and high legal compliance costs degrade RWD accessibility and usability for research (33).

### 3.2. Separate consent

The primary obstacle to processing RWD is obtaining the PI subject’s consent. This requirement resolves the ethical data quandary associated with RWS involving humans. It is rooted in the right to informational self-determination, which aims to increase individual control over one’s PI. This right is guaranteed in both the GDPR (34) and U.S. California Consumer Privacy Act (35) and is also recognized in China’s PIPL.

Under PIPL, PI processors are obligated to obtain specific, informed consent from PI subjects regarding their PI handling activities. The individual should voluntarily and explicitly give such consent on a fully informed basis. As for sensitive PI processing, the PIPL sets higher standards concerning informed consent and notification duties; in such instances, separate consent is mandatory for certain processing activities.

The PIPL does not define “separate consent.” However, the semantic interpretation clearly indicates that it does not permit “broad/blanket consent.” This means that RWS researchers must inform personal data subjects about the specific purpose, necessity, and impact of PI processing for specific research projects and obtain explicit consent from each individual. This process can be difficult, time-consuming, and costly. However, many medical breakthroughs rely on large-scale data collection of sensitive MHDs (36). Without broad access to and use of such data, RWS is not feasible. Ergo, PIPL’s separate consent requirement limits big data analytics as it reduces the volume of data available for analysis.

### 3.3. Data desensitization

Data desensitization technologies, such as anonymization and de-identification, are critical to ensuring the legal compliance of RWD processing. Anonymity exists on a continuum from complete anonymity to fully identifiable data (37); de-identified data lies somewhere in the middle.

Anonymized PI is outside the regulatory scope of the PIPL, so its use would not violate the PIPL. However, the anonymization of rich MHDs may limit data linkage possibilities and exclude information crucial to RWS, reducing the data’s usefulness (38). For example, the anonymized data set of “John Doe, 14 years old” could be “Male, 10–20 years,” which distorts the data.

In contrast, de-identification seeks to preserve as much of the underlying data as possible, as a precursor to anonymization (39). According to the U.S. Health Insurance Portability and Accountability Act (*HIPAA*) de-identified *PI* under PIPL is not identical to de-identified protected health information. Instead, it is consistent with the GDPR’s definition of pseudonymization. The de-identification technology separates data from personal identity by removing identifying attributes. This action reduces the significant identifying risks to an acceptably small level. However, the de-identified RWD still falls into PIPL’s protection scope since the re-identification risk remains. Therefore, the current PIPL de-identification rule is too simplistic to support RWS since it neither clarifies the criteria for lawful de-identification nor establishes when de-identified PI can be considered “anonymous.”

### 3.4. PIA and cross-border data transfer

Real-world data-based study researchers should conduct a prior PI-PIA (Table 2). A PI-PIA must be conducted if PI processors want to transmit RWD abroad. This institution is somewhat similar to the “data protection impact assessment” required under GDPR, which helps negate risk.

**Table 2:** The content of the PI-PIA (article 56 PIPL).

The PI-PIA includes:	
1	The lawfulness, legitimacy, and necessity of the purpose and method of handling PI.
2	The impact on individuals’ rights and interests and the security risks involved.
3	The legality, effectiveness, and appropriateness of protective measures taken in relation to the level of risk.

PI-PIA, Personal information protection impact assessment.

The RWD processing risk is not static or binary. Instead, it exists on a spectrum that changes with the processing scenario. The PI-PIA conducts a case-by-case, context-based analysis to evaluate the risk degree and the effectiveness of the protective measures taken, which structurally embeds the legal and ethical considerations of regulators into the PI processing. It introduces legal obligations to force PI processors to “identify, assess, and ultimately manage the high risks to rights and freedoms” (40) posed by PI processing beforehand, ultimately shifting the PI protection model from an ex-post regulation to an ex-ante prevention model based on risk management.

Legitimate cross-border data transfer (CBDT) requires the PI processor to pass a security assessment organized by the Cyberspace Administration of China (CAC), obtain a PI protection certification from a body recognized by the CAC, or sign a standard contract formulated by the CAC with the overseas recipient (article 38 PIPL). In addition, the PIPL imposes stricter restrictions on CBDT than most data protection laws by requiring certain entities to comply with PI localization obligations (article 40 PIPL). To carry out CBDT, the PI processor must also notify PI subjects of the specific information about the CBDT and obtain their separate consent (article 39 PIPL). Unlike the GDPR, the PIPL does not have an “adequacy decision” or “appropriate safeguards” mechanism for CBDT or various exceptions for specific situations from the prohibition. These requirements have made CBDT challenging to execute.

## 4. Discussion

This article has demonstrated the legal dilemmas of conducting RWS in China, mainly the compliance hurdles related to PI protection. The primary factor impeding effective data utilization and weakening individual rights and interests protection is the lag of relevant legislation and regulations in data governance in public health. This article proposes several countermeasures to strike a balance between data utility and privacy protection.

### 4.1. Introduce the meta-consent model

In RWS based on big data, researchers can uncover patterns of human physiology and pathology by comparing data from different races, disease spectrums, and environments. These data inevitably contain sensitive PI. Strict consent obligations hinder such data collection, limiting opportunities for RWS. Accordingly, a consent model must be developed to address the potential conflict between active data use and informed consent principles.

The level of privacy risk depends upon how the PI is used in varying scenarios. Thus, consent models should adjust accordingly (41). Legislators should construct a dynamic, context-based meta-consent model for scientific research to enable participants to give either specific or broad consent depending on personal preferences for the secondary use of their PI (42). Compared to broad consent, which may not meet ethical requirements in some medical studies involving humans, the meta-consent model better balances the legal and ethical obligations of the researcher. It suggests, for example, establishing a web-based portal or application that allows individual research participants to dynamically provide blanket or specific consents for different personal data types based on their preferences, depending on the different RWS project categories they are involved in (43). Specifically, the research participant might choose to give blanket consent for RWS in the public interest, to provide separate consent for commercial RWD use, or to refuse consent to transfers of personal data across borders; moreover, participants can modify their initial choices (44–48). Active participation in the tiered-consent process, which improves interactions between healthcare providers and patients (49), along with increased transparency regarding PI use, are the main advantages of this model. Opponents argue that its implementation costs are higher than broad consent or dynamic consent model (44, 50). However, implementing meta-consent would reduce the average costs of obtaining consent from PI subjects compared to the current specific, separate informed model while improving the availability of MHDs in RWS and respect for participants’ autonomy. Therefore, this is a pragmatic approach.

### 4.2. Clarify de-identified criteria

To overcome the legal barriers in RWD processing, we recommend regulators adopt a pragmatic, risk-based approach to clarify the criteria for “adequately” de-identifying PI in different scenarios to preserve as much data utility as possible. China’s regulators should consider adopting HIPAA-like privacy rules, which allow sharing and using health data without informed consent as long as the data has been de-identified by expert determination or the safe harbor method. To prevent the weakening of de-identification criteria due to rapid technological advances, PI processors should be contractually prohibited from re-identifying PI and be held liable for non-compliance.

Medical health data have unique public interest attributes (25). RWS that processes large amounts of MHDs can promote the prevention and control of infectious diseases, drug development, and more, which greatly enhance public health interests. Strict de-identified criteria is more biased toward PI protection and partially sacrifices the data utilization, given that different data types have different requirements for protection and utilization. This suggests that the homogeneous de-identified criteria cannot be simultaneously applied to the de-identification needs of different PI types, such as financial data, sensitive PI such as religious beliefs or sexual orientation, and MHDs. On this basis, in particular, the de-identification of MHDs should be regulated. Specifically, China should consider enacting separate regulations on MHDs in addition to the PIPL. Meanwhile, the government should authenticate specialized institutions with ultimate responsibility for MHD processing and de-identification (51). This makes it easier for research institutions to choose suitable de-identification service providers; moreover, it reduces their compliance risks and encourages them to share research data, ultimately improving the availability and quality of MHDs, while balancing data protection and utilization.

### 4.3. Balancing interests

Maximizing RWD potential and protecting patients’ PI are critical values. If they are in conflict, the regulatory framework must be adjusted to balance them.

The PIPL improves accountability for protecting PI with strict regulations like those on CBDT. However, it does not sufficiently balance research promotion with the global trend toward responsible data sharing. We cannot ignore that the MHDs is not only a matter of patient welfare but also a matter of public interest. The EU recently introduced a new concept of data altruism in the Data Governance Act and published a proposal for the European Health Data Space to improve MHDs availability. In contrast, China lacks similar legislation and a unified, effective PI protection agency with independent authority, which may place China at an international disadvantage for RWD use.

China’s National Standard “Information Security Technology—Guide for Health Data Security 2020” (GHDS) introduced the concept of a “limited data set,” which stipulates that de-identified data could be used or disclosed without the PI subject’s consent when used for scientific research, medical/health education, and public health purposes. However, the GHDS is a recommended national standard without legal force and inconsistent with current PIPL rules. To address this, reasonable proportionate legal derogations and exceptions for processing PI should be enacted for scientific and medical purposes, as well as for the public good (52). We urge legislators to revise the PI regulatory framework to balance “risk control” and “industry promotion” (53). The NMPA could create a negative list of PI processing practices for RWS to protect citizens’ PI rights and avoid unclear regulations. Likewise, international organizations (e.g., WHO) should lead in strengthening cooperation and collaboration among member countries to protect MHDs within multilateral frameworks.

### 4.4. Conclusion

Information technology-derived RWS is promoting a new round of digital revolution in the health field. At the same time, it brings PI challenges which urgently need to be solved. To promote the RWS to support public health efforts, we should clarify the regulatory model of utilitarianism of RWD in public health care and resolve the tension between active utilization and strict data protection. China’s legislation ought to encourage the active use of data in public health care through the introduction of the flexible meta-consent model, the adoption of a risk-based approach to interpreting and clarifying the de-identifying PI criteria, and weakening the current strict regulatory framework by introducing other supplementary mechanisms to balance the protection of PI and conflict of public value.

## Data availability statement

The original contributions presented in the study are included in the article/supplementary material, further inquiries can be directed to the corresponding author.

## Author contributions

YY: Conceptualization, Data curation, Supervision, Writing – original draft, Writing – review & editing. FY: Conceptualization, Data curation, Supervision, Writing – original draft, Writing – review & editing.

## Funding

The author(s) declare financial support was received for the research, authorship, and/or publication of this article. This work was supported by National Social Science Fund of China Youth Project under Grant (19CFX063). The funder was not involved in the study design, collection, analysis, interpretation of data, the writing of this article, or the decision to submit it for publication.

## Conflict of interest

The authors declare that the research was conducted in the absence of any commercial or financial relationships that could be construed as a potential conflict of interest.

## Publisher’s note

All claims expressed in this article are solely those of the authors and do not necessarily represent those of their affiliated organizations, or those of the publisher, the editors and the reviewers. Any product that may be evaluated in this article, or claim that may be made by its manufacturer, is not guaranteed or endorsed by the publisher.
